# Polar mesoporous zinc sulfide nanosheets encapsulated in reduced graphene oxide three-dimensional foams for sulfur host

**DOI:** 10.1038/s41598-020-62037-4

**Published:** 2020-03-24

**Authors:** Limin Mao, Fei Wang, Jian Mao

**Affiliations:** College of Materials Science and Engineering, Sichuan University, Chengdu, 610065 China

**Keywords:** Batteries, Batteries, Two-dimensional materials

## Abstract

Lithium-sulfur (Li-S) batteries exhibit the high specific capacity and energy density, but prevented by the low coulombic efficiency and weak cycle life. Herein, we fabricate reduced graphene oxide (r-GO) three-dimensional (3D) foams encapsulating polar mesoporous zinc sulfide (ZnS) nanosheets and subsequently utilize the ZnS/r-GO foams to load sulfur (ZnS/r-GO/S) as cathodes for improving the performance of Li-S batteries. The mesoporous diameter of the ZnS nanosheets is approximately 10~30 nm and lots of pores in the 3D foams are observed. The porous structure provides abundant sites to adsorb and accommodate sulfur species. The cathode of the ZnS/r-GO/S exhibits 1259 mA h g^−1^ of initial capacity and 971.9 mA h g^−1^ of the reversible capacity after 200 cycles at 0.1 C (1 C = 1675 mA g^−1^). At 1 C, it still exhibits the tiny capacity decay rate of 0.019% per cycle after 300 cycles. This work may be adopted to combine the nonpolar and polar materials as a 3D network structure for high-performance Li-S batteries.

## Introduction

Lithium-sulfur (Li-S) batteries, as the most promising next-generation rechargeable batteries, possess an ultrahigh theoretical specific capacity of 1675 mA h g^−1^ and excellent theoretical energy density of 2600 W h Kg^−1^, up to five times greater than commercial LIBs (387 W h Kg^−1^ for LiCoO_2_/C battery)^1,2^. In addition, sulfur element is abundant, low-cost and environmentally inert^3^. Therefore, Li-S batteries are supposed to most promising candidate energy device to satisfy the request of electronic equipment and the demand of preferable pure electric vehicles^4^. However, Li-S batteries are still impeded by the low coulombic efficiency and the poor cycling stability, due to three factors: (1) the insulation of sulfur and its final product Li_2_S/Li_2_S_2_, (2) large volume change during charge and discharge, and (3) the shuttling effect. The insulation of sulfur and Li_2_S_2_/Li_2_S limits the redox reaction, causing the low utilization of active materials. Consequently, uniform dispersion of sulfur and good electrical contact with conductive matrixes are essential^5^. Volume change of sulfur under alloying is approximately 80%, making the electrode broken easily^6^. Many porous materials and internal void nanoarchitectures were designed to accommodate the expansion^7^. Shuttling effect is the critical issue in Li-S batteries, which causes low charge efficiency and poor cyclic stability. Many researches manifested that physical trapping by porous nanostructure and chemical affinity by polar materials can hinder shuttling effect availably^8,9^.

Up to now, sulfur was loaded into various materials with diverse morphologies, including carbonaceous materials^10–12^, conductive polymers^13–15^, metal oxides^16,17^, and metal sulfides^18,19^, etc. Carbonaceous frameworks were popular on account of their good conductivity and abundant morphologies, such as meso/microporous^20^, fiber-like and foam-like structure^21,22^. Graphene with high surface area, chemical stability, mechanical strength and flexibility, was utilized as a common energy material^23^. Nevertheless, the graphene is nonpolar nature, so polysulfides are easy to escape from the surface of graphene^24^. Oxides and sulfides are better in restraining polysulfides migrating due to their polarity^19,25^. Metal sulfides have a strong affinity for polysulfides caused by strong sulfiphilic property, but suffering from low conductivity^26^. Therefore, studies in combination the graphene and polar materials to achieve the good conductivity and reduce polysulfides shuttling were prominent^27^.

**Figure 1:**
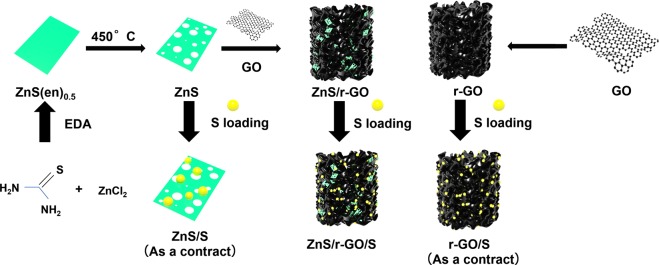
Schematic illustration showing the preparation ZnS/S, r-GO/S and ZnS/r-GO/S foams composites.

Herein, we designed a three-dimensional (3D) porous foams, constructed by high conductive reduced graphene oxide (r-GO), to encapsulate polar mesoporous ZnS nanosheets by a facile water bath method. Then, the ZnS/r-GO foams loaded sulfur as composite electrodes for high-performance Li-S batteries. The r-GO foam and ZnS nanosheets were used to load sulfur as contrasted. The schematic illustration was shown in Fig. 1. The mesoporous ZnS nanosheets were synthesized by sintering zinc sulfide (C_2_H_4_(NH_2_)_2_)_0.5_ (ZnS(en)_0.5_) precursor with a superior catalysis^28,29^. The strong interaction with soluble polysulfides and catalytic activation of ZnS makes sure the sulfur utilization and stable cycle performance^30,31^. The 3D porous r-GO network possessed high conductivity, promoting the rapid diffusion of Li^+^, but also providing the abundant sites to accommodate the sulfur species and release the expansion. The cathode of the 3D ZnS/r-GO/S foams represented excellent electrochemical performance, such as the high initial capacity of 1259 mA h g^−1^ and a stable capacity retention of 971.9 mA h g^−1^ after 200 cycles at 0.1C (1C = 1675 mA g^−1^), remarkable coulombic efficiency of close to 100% as well as low capacity fading rate of 0.019% per cycle after 300 cycles at 1C.

## Results and Discussion

**Figure 2:**
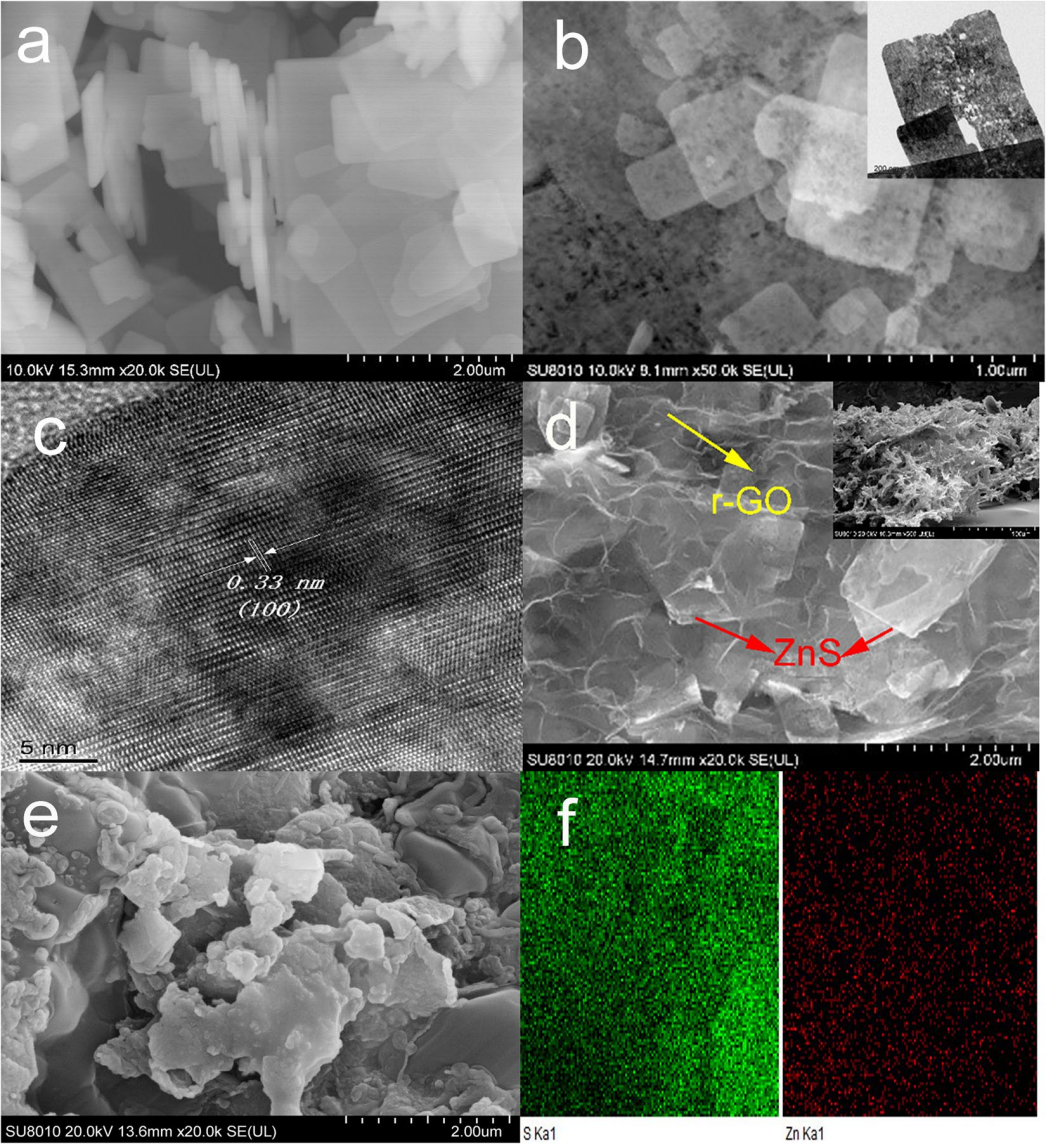
SEM and TEM images of the samples. (**a**) ZnS (en)_0.5_, (**b**) mesoporous ZnS, the insert is TEM image of mesoporous ZnS. (**c**) HRTEM image of mesoporous ZnS, (**d**) SEM images of ZnS/r-GO, the insert is the cross section SEM of ZnS/r-GO. ZnS nanosheets were guided by red arrows, and r-GO was guided by yellow arrows. (**e**) SEM image of ZnS/r-GO/S. (**f**) the EDS mapping of (**e**).

The SEM and TEM, HR-TEM images were shown in Fig. 2. The ZnS(en)_0.5_ was composed of large quantities of smooth nanosheets with the thickness of 60~100 nm, and the large range of a few to a dozen micrometers in lateral dimensions (Fig. 2a).The mesoporous ZnS succeeded to the similar sheet nanostructure in Fig. 2b, and the porosities were triggered by the decomposition of ethylenediamine (EDA)^32^, which could provide more sites to adsorb and catalyze polysulfides. The insert TEM image showed that the pore diameter was approximately 10~30 nm. HRTEM observation in Fig. 2c showed well-resolved lattice planes with a lattice spacing of 0.33 nm (Corresponding to (100) plane of ZnS). The SEM of the ZnS/r-GO foams (Fig. 2d) indicated that the ZnS nanosheets were encapsulated in the r-GO foams. The insert was the cross section SEM of ZnS/r-GO, manifested the 3D porous structure of the prepared composite, which played a vital role in accommodating sulfur and confinement physically to polysulfides. The loaded sulfur was distributed in the ZnS/r-GO composite as Fig. 2e. The EDS mapping (Fig. 2f) of Zn and S elements elucidated the uniform distribution of sulfur and the ZnS nanosheets.

**Figure 3:**
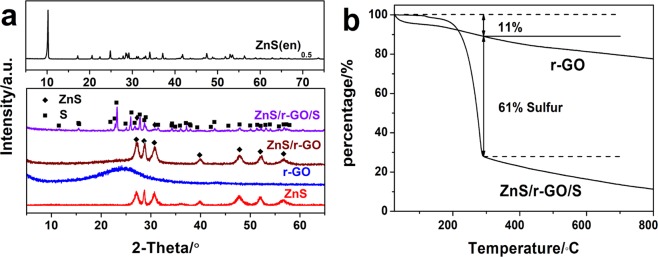
XRD patterns and TG curves of the samples. (**a**) XRD patterns of ZnS(en)_0.5_, ZnS, r-GO, ZnS/r-GO and ZnS/r-GO/S. (**b**) TG curves of ZnS/r-GO/S and r-GO.

The XRD patterns of these samples were shown in Fig. 3a. The ZnS(en)_0.5_ was indexed in the orthorhombic system, consistent with the previous studies^29,33^. After 450 °C annealing, the EDA was decomposed and the product was transformed to wurtzite ZnS^34^. Compared with the XRD pattern of the r-GO, the ZnS/r-GO composite had no obvious peak at approximate 26°, corresponding to (002) plane of r-GO. It probably resulted from the ZnS nanosheets preventing the restocking of the r-GO nanosheets^35^. The sulfur of the ZnS/r-GO/S was indexed to the orthorhombic system (JCPDS card 08-0247).

The thermogravimetry (TG) curves of the ZnS/r-GO/S composite and r-GO in N_2_ atmosphere were shown in Fig. 3b. The plummet of the ZnS/r-GO/S composite mainly due to the evaporation of the sulfur. The curve of the r-GO presented near-linear decline, which may cause by the decomposed of the unreduced oxygen functional group. Compared with the curve of the r-GO, the decline of the ZnS/r-GO/S composite was comprised of 11% and 61% resulted from the r-GO and sulfur, respectively. After heated to over 300 °C, the almost parallel curves also indicated that the declined weight of the ZnS/r-GO/S composite influenced by the r-GO.

**Figure 4:**
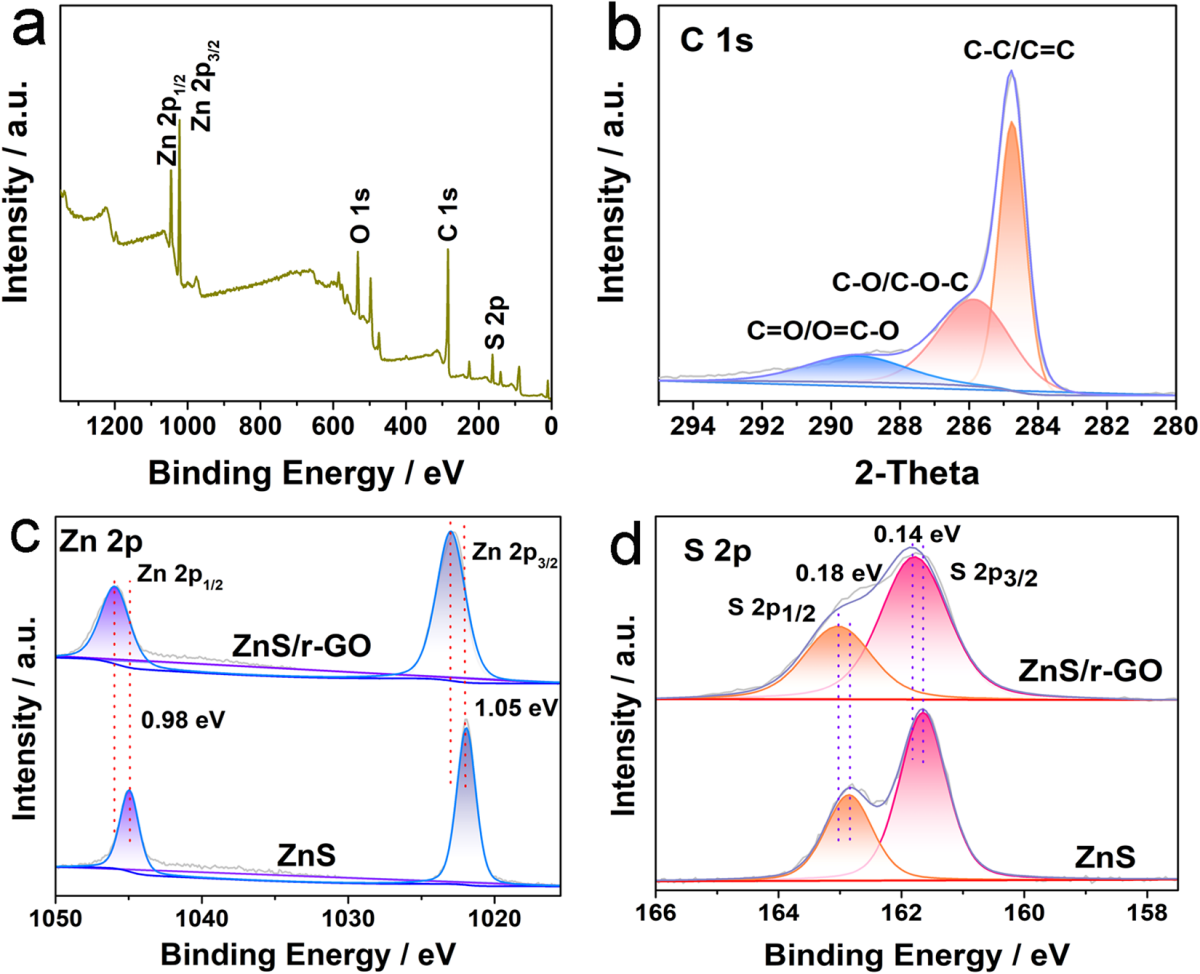
XPS spectra of the ZnS/r-GO and ZnS composite. (**a**) Wide spectrum of ZnS/r-GO. (**b**) C 1 s spectrum of ZnS/r-GO. (**c**,**d**) S 2p and Zn 2p spectra of ZnS/r-GO composite and ZnS nanosheets.

X-ray photoelectron spectroscopy (XPS) was conducted to further investigate the chemical states of the ZnS/r-GO foams in Fig. 4. The wide spectra (Fig. 4a) showed the presence of C 1 s, O 1 s, Zn 2p and S 2p peaks. The C 1 s spectrum (Fig. 4b) exhibited a noteworthy peak at 284.5 eV, ascribed to C-C/C=C. Other peaks located at 285.9 and 289.3 eV were ascribed to C-O/C-O-C and O=C-O/C=O, respectively. Figure 4c,d showed the S 2p and Zn 2p spectra of ZnS/r-GO composite and ZnS nanosheets. The peak separation of the peaks at 1022.5 and 1045.9 eV in Zn 2p spectrum of ZnS/r-GO composite was 23.0 eV, which was originated from the Zn 2p_3/2_ and Zn 2p_1/2_ of ZnS^36^. Compared with the ZnS nanosheets, there was a positive shift of 1.05 and 0.98 eV for Zn 2p_3/2_ and Zn 2p_1/2_ of ZnS/r-GO composite, respectively. Correspondingly, the S 2p could be deconvoluted into the peaks at 162.0 and 162.9 eV, assigned to S 2p_3/2_ and S 2p_1/2_ of ZnS. The peaks in S 2p spectrum of ZnS/r-GO composite also shifted to the higher binding energy. These indicated the electronically coupled between ZnS and r-GO^37^.

**Figure 5:**
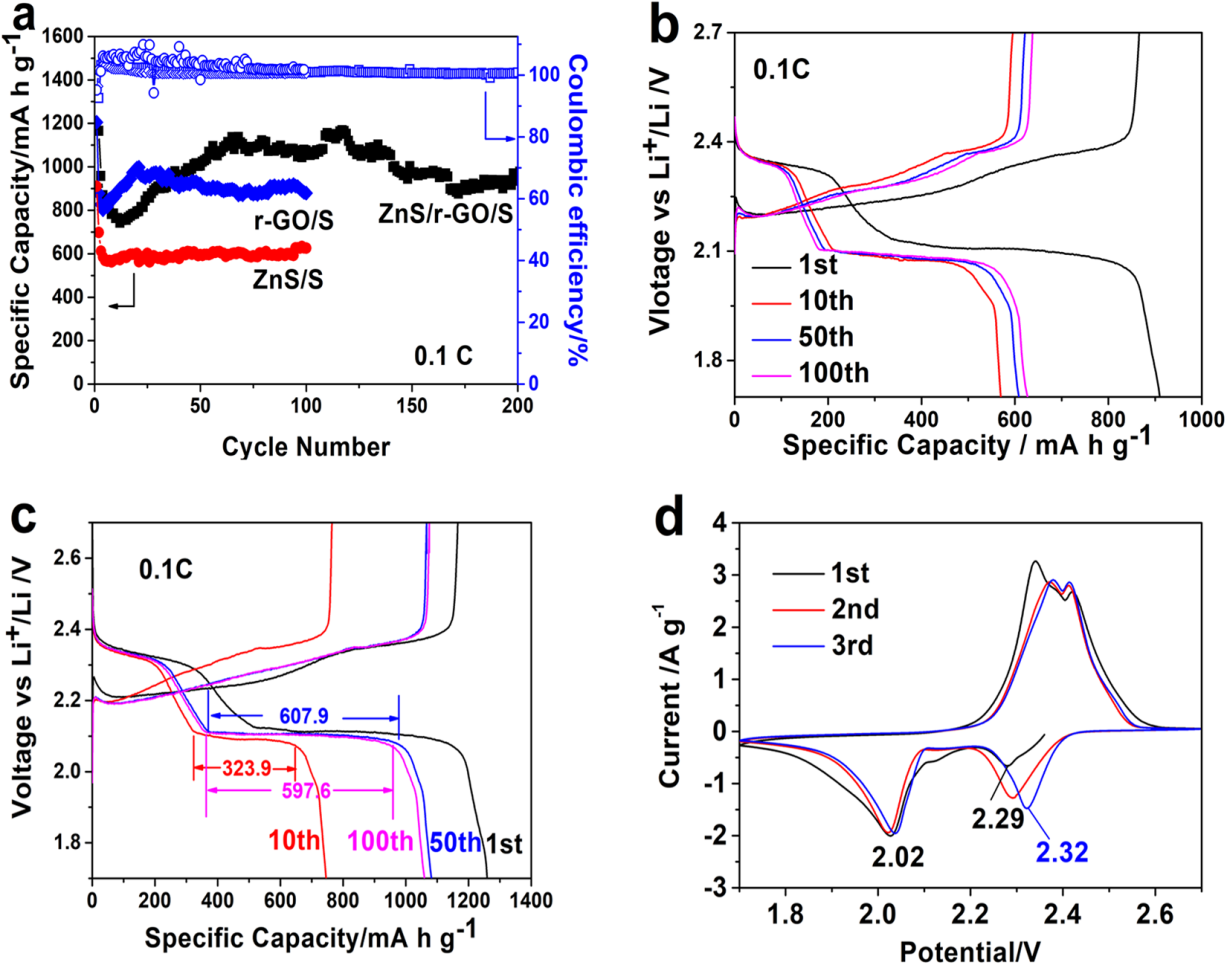
Electrochemical performance of ZnS/r-GO/S, r-GO/S and ZnS/S electrodes. (**a**) Cycle performance at 0.1 C of ZnS/r-GO/S, r-GO/S and ZnS/S electrodes, respectively. (**b**,**c**) Galvanostatic charge/discharge profiles of ZnS/S and ZnS/r-GO/S cathode at 0.1 C. (**d**) CV curves of ZnS/r-GO/S electrode.

The electrochemical performance of ZnS/r-GO/S and ZnS/S cathodes was displayed in Fig. 5. The cycle performance of ZnS/r-GO/S, r-GO/S and ZnS/S at 0.1 C were exhibited in Fig. 5a. The initial specific capacities of ZnS/r-GO/S, r-GO/S and ZnS/S were 1259.4, 1205.3 and 910.2 mA h g^−1^, respectively, retained the reversible capacity of 1059.2, 879.0 and 626.6 mA h g^−1^ after 100 cycles. The ZnS/S cathode exhibited a lower initial specific capacity and dramatic capacity fading in the second cycle. However, between 3 and 100 cycles, the specific capacity maintained stable, suggesting that the ZnS had a strong affinity and good catalytic activity to polysulfides. The almost coincident galvanostatic charge/discharge curves of the ZnS/S cathode at 10th, 50th and 100th (Fig. 5b) confirmed the stable cycle. Because of the high specific surface area and high conductivity of the r-GO 3D network, the ZnS/r-GO/S and r-GO/S exhibited a higher initial specific capacity. In the first few cycles, both ZnS/r-GO/S and r-GO/S showed a severe capacity fading, after that, the specific capacity increased gradually. The reasons were as follows: as the electrolyte penetrated, the sulfur loaded in the 3D foams would gradually react with Li^+^; and for the ZnS/r-GO/S cathode, owing to the strong adsorption and catalysis of ZnS^31,38^, the mesoporous ZnS encapsulated in 3D foams would gradually adsorb polysulfides dissolving in electrolyte and catalyze the conversion of sulfur redox^30^. The above was also proved by the manifested second charge/discharge voltage profiles at the 50th and 100th cycle of the ZnS/r-GO/S cathode, which were longer than that at the 10th cycle with the similar first voltage profiles (Fig. 5c). The 50th and 100th cycle displayed specific capacities of 607.9 and 597.6 mA h g^−1^ at the second plateau, nonetheless the second plateau of the 10th only discharged 323.9 mA h g^−1^. The synergistic effect of the high conductive 3D network and strong-sulfiphilic ZnS made the ZnS/r-GO/S cathode deliver more excellent electrochemical performance. Even after 200 cycles, the reversible capacity of the ZnS/r-GO/S was 971.9 mA h g^−1^.

CV curves of the ZnS/r-GO/S were recorded at a scan rate of 0.1 mV s^−1^ in the potential range of 1.7~2.7 V (vs. Li/Li^+^). The typical redox reaction of sulfur cathodes was presented in Fig. 5d. In the first cycle, two reduction peaks at 2.29 V and 2.02 V were observed, ascribing to the formation of long-chain lithium polysulfides and the further reduction to the short-chain lithium sulfides, respectively. In the third cycle, the reduction peak at 2.29 V shifted to higher potential (2.32 V). It demonstrated faster electrochemical kinetics and lower cell polarization, meaning the better cycle reversibility of the ZnS/r-GO/S cathode^11^.

**Figure 6:**
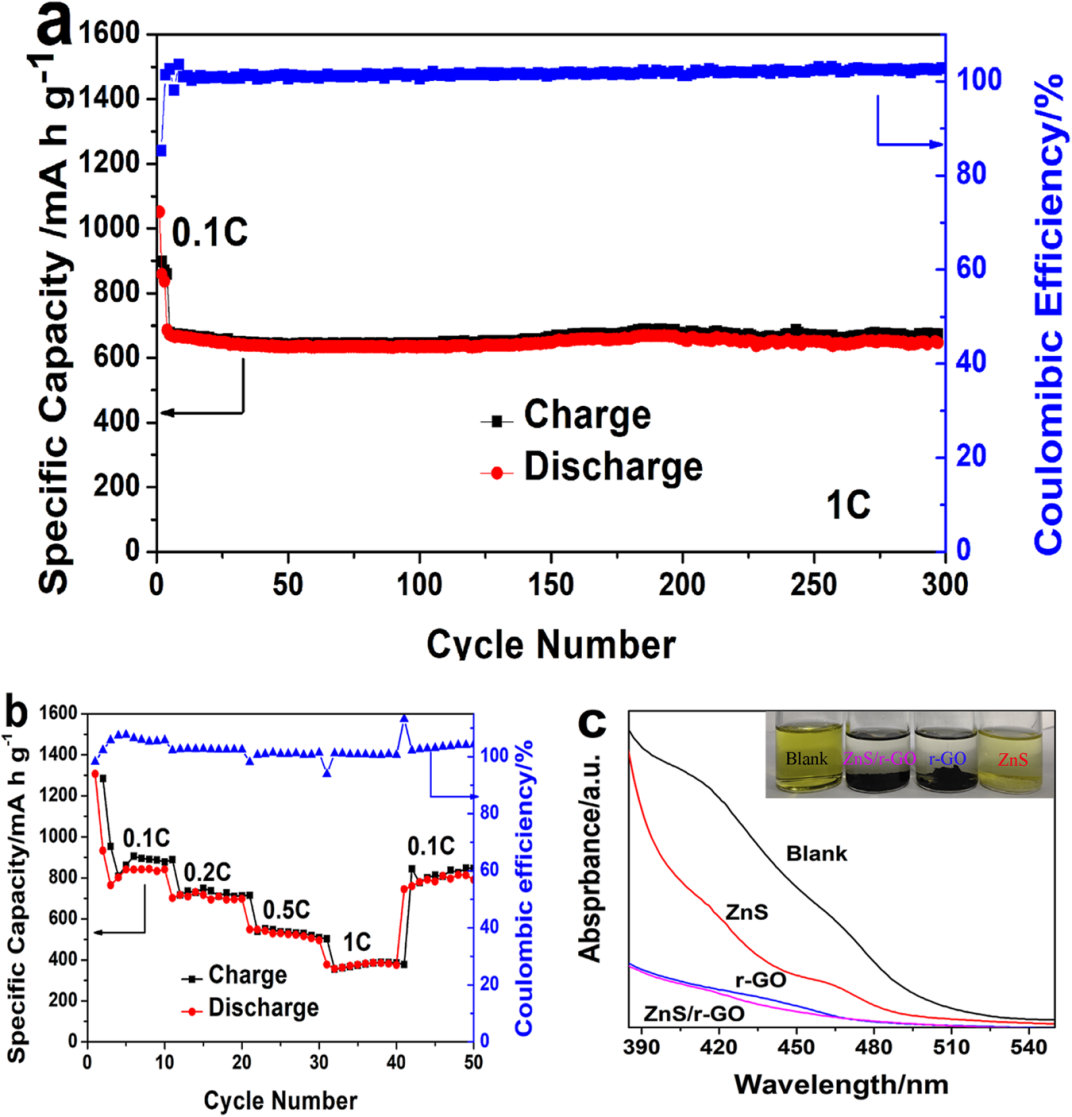
Electrochemical performance and Polysulfides adsorption. (**a**) Long-term cycling performance of ZnS/r-GO/S at 1 C after activation in first three cycles at 0.1 C. (**b**) Rate capacity of ZnS/r-GO/S, (**c**) UV-vis spectra of the Li_2_S_6_ solution with blank, ZnS, r-GO and ZnS/r-GO. (Inset photograph of visualized adsorption of Li_2_S_6_ on ZnS, r-GO and ZnS/r-GO with same mass).

The long-term cycling performance of the ZnS/r-GO/S at 1 C after activation in first three cycles at 0.1 C was shown in Fig. 6a. After 300 cycles, a discharge specific capacity of 646.3 mA h g^−1^ with ~100% coulombic efficiency was remained, corresponding to a low capacity fading of 0.019% per cycle. The rate capacity (Fig. 6b) of the ZnS/r-GO/S was measured at different current rate in the potential range of 1.7~2.7 V (vs. Li/Li^+^). When the rate turned back to 0.1 C after each 10 cycles at 0.1 C, 0.2 C, 0.5 C and 1 C, a discharge capacity of approximately 800 mA h g^−1^ was recovered, indicating the stable rate performance.

The Li_2_S_6_ adsorption was measured to visually observe the affinity of the ZnS/r-GO, r-GO and ZnS to polysulfides, and the UV-vis spectra were carried out to test the concentration of Li_2_S_6_ solutions (Shown in Fig. 6c). In the inset photograph, the noticeable fade in color of the Li_2_S_6_ solutions was observed after adding the ZnS/r-GO and r-GO  due to the disproportionation of the polysulfides^39^ and the adsorption of samples. The affinity of r-GO to plysulfides may be caused by the higher specific area. On the other hand, the change in color of the Li_2_S_6_ solution of ZnS treatment was lighter. In the UV-vis spectra, a strong absorbance was observed at 420 and 465 nm in fresh Li_2_S_6_ solution, which ascribed to the adsorption peaks of the Li_2_S_6_. The absorbance of ZnS was declined slightly, and the peaks were still visible. For the solutions after the r-GO and ZnS/r-GO treatment, the peak almost disappeared, indicating that the r-GO and ZnS/r-GO foams had strong affinity to Li_2_S_6_. From the UV spectra, the ZnS/r-GO foams combining the polarity of ZnS and the high specific surface area of r-GO exhibited the best absorptivity to the Li_2_S_6_.

In summary, the 3D foams comprised by polar mesoporous ZnS nanosheets and r-GO was fabricated, then the sulfur was loaded into the foams as the Li-S batteries cathodes. The pore diamter of the mesoporous ZnS nanosheets was 10–30 nm and the ZnS/r-GO composite possessed lots of pores. The 3D ZnS/r-GO foams loaded sulfur exhibited excellent electrochemical properties, initial specific capacity of 1259 mA h g^−1^ and a reversible capacity of 971.9 mA h g^−1^ after 200 cycles at 0.1 C, due to the polar adsorption to polysulfides by the mesoporous ZnS and the excellent Li^+^ or electrons diffusion of the r-GO foams. This work may provide a way to combine the nonpolar and polar materials as a 3D network structure for high-performance Li-S batteries.

## Methods

### Preparation of the ZnS(en)_0.5_, mesoporous ZnS nanosheets, r-GO and ZnS/r-GO 3D foams

EDA as solvent, the mesoporous ZnS nanosheets were synthesized by solvothermal and subsequent calcination^28^. 2 mmol ZnCl_2_ and 4 mmol CS(NH_2_)_2_ were mixed uniformly, then dissolved in 60 ml EDA with ultrasonic for 30 min. The solution was quickly poured into 100 mL autoclave, solvothermal at 180 °C for 12 h. After centrifuging and cleaning, drying at 60 °C for hours, the ZnS(en)_0.5_ precursor was obtained. The mesoporous ZnS nanosheets were got by calcinating the ZnS(en)_0.5_ at 450 °C for 30 min in air. The ZnS/r-GO 3D foams were synthesized by a facile excess ascorbic acid reduction method and the mass ratio of the ZnS and GO was 1:1. The mixture in distilled water was sonicated for 10 min, and then heated at 90 °C for 2 h in a water bath. The freeze drying was used to keep the porous structure. The r-GO foam was prepared by the same method of ZnS/r-GO for a contract.

### Preparation of the ZnS/S, r-GO/S and ZnS/r-GO/S composites

The ZnS/S, r-GO/S and ZnS/r-GO/S composites were prepared by the conventional melt-diffusion method. Typically, sulfur powders and the samples (ZnS, r-GO or ZnS/r-GO) in a mass ratio of 3:2 were homogenized by ground in an agate mortar. The mixtures were sealed in 25 mL Teflon-lined stainless-steel autoclave with argon in glove box, maintained at 155 °C for 12 h.

### Characterization

SEM images were obtained with a scanning electron microscope. TEM and high-resolution TEM (HRTEM) images were performed by a transmission electron microscopy at 200 KV. The X-ray diffraction measurements were carried out using an X-ray powder diffractometer, performed in transmission geometry with Cu K_α1_ radiation (λ = 1.5418 Å). The samples were measured at the sweep rate of 5° min^−1^. X-ray photoelectron spectroscopy (XPS) measurements were performed on a Kratos XSAM 800 (UK). UV-vis spectra of the Li_2_S_6_ solutions exposed to different adsorbents were collected on a UV-vis spectrometer. The sulfur contents of composites were determined using thermo-gravimetry analyses (TGA) on a DTG-60 from the room temperature to 800 °C with a heating rate of 10 °C min^−1^ in Ar.

### Polysulfides adsorption tests

Lithium polysulfide (Li_2_S_6_) solution was prepared as the literature reported^19^. The stoichiometric ratio amounts of sublimed sulfur and lithium sulfide (Li_2_S) with a molar ratio of 5:1 were dissolved in 1:1 (v/v) DOL/DME, then sealed in an argon-filled glove box, stirred at 60 °C for 48 h in the ambient environment. 10 mg the ZnS, r-GO or ZnS/r-GO was added in an 8 ml Li_2_S_6_ solution (1 mmol L^−1^) and stewed for 24 h. The UV-vis spectra were tested to analyze the adsorption ability of the ZnS, r-GO or ZnS/r-GO for Li_2_S_6_.

### Cell assembly and electrochemical measurements

The electrochemical tests were performed using a coin type 2032 half-cell with lithium metal (purity 99.95%, 0.6 mm thick and 15.8 mm in diameter) as counter and reference electrodes, and the polypropylene films (Ceglard 2400) were as separators. The electrode slurry was composed of 80 wt. % active materials (the ZnS/S, r-GO/S or ZnS/r-GO/S), 10 wt. % acetylene black and 10 wt. % polyvinylidene fluoride (PVDF), moderate n-methyl-2-pyrrolidinone (NMP) as solvent. The active materials and acetylene black, PVDF were ground uniformly in an agate mortar for 1 h. The slurry was coated onto carbon paper (thickness of 20 μm) with subsequent heating at 60 °C for 24 h in vacuum. The composite carbon paper was then punched into disk with 14.2 mm in diameter to be used as cathodes. The areal sulfur loadings were ranged at 1.3~2.2 mg cm^−2^. 1 mol L^−1^ lithium bistrifluoromethyl sulfimide (LiTFSI) in DME and DOL (1:1 v/v) with 2 wt. % LiNO_3_ was used as the electrolyte. The galvanostatic charge/discharge performance of the cells was tested on a multichannel battery tester (LANDCT) in the potential range of 1.7~2.7 V vs. Li^+^/Li electrodes at room temperature. Cyclic Voltammetry (CV) measurements were performed at a scan rate of 0.1 mV s^−1^ on an electrochemical workstation. Applied currents and specific capacities were calculated on the basis of the mass ratio of S in the cathodes.
